# Connections Between Gene Polymorphism and Fetlock and Hock Measurements in Polish Sport Horses

**DOI:** 10.3390/ijms26199645

**Published:** 2025-10-02

**Authors:** Dorota Lewczuk, Maria Wypchło, Mateusz Hecold, Roma Buczkowska, Agnieszka Korwin-Kossakowska

**Affiliations:** 1 Institute of Genetics and Animal Biotechnology PAN, ul. Postępu 36A, 05-551 Magdalenka, Poland; maria.wypchlo@nio.gov.pl (M.W.);; 2 Maria Sklodowska-Curie National Research Institute of Oncology, ul. W. K. Roentgena 5, 02-781 Warszawa, Poland; 3 Faculty of Veterinary Medicine, Warsaw University of Life Sciences, ul. Nowourysnowska 166, 02-787 Warszawa, Poland

**Keywords:** sport horse health, bone development, genes, bone size measurement

## Abstract

Finding the causative mutations for musculoskeletal system development and health status is of a higher priority for all sport horse breeders’ associations. Of the regulating proteins involved in animal ossification, 15 gene polymorphisms were chosen to be identified as connected with the nine fetlock and 14 hock bone structures measurements of 198 horses. All measurements were taken using X-rays of the limbs, which were available at the beginning and end of the horse training. The analysis of variance (GLM, SAS program) was performed taking into account identified training and horse-connected characteristics, and gene polymorphism. The larger size of the bone structure was achieved in the fetlock for the heterozygotes of *COL9A2*, *AOAH1*, *BMPER*, *HYAL3*, and *ELMO1*. The heterozygotes were superior to homozygotes in the hock for the following genes: *COL9A2*, *HYAL3*, *ANLN*, and *HYAL1*. The lower homozygote values were obtained for GG in *CPVL* in fetlock measurements, TT for *HYAL3* in fetlock, TT for *ANLN* in fetlock, CC for *FRZB* in the hock, TT for *MATN* in the hock, and TT for *COL5A2* in the hock than their opposite homozygote and heterozygote variants. *COL9A2* and *HYAL3* are expressed in the same way for most of the bone structures in both joints.

## 1. Introduction

The musculoskeletal system is extremely significant for the sport horse’s performance. Finding the causative mutations for its development and health seems to be of a higher priority for all sport horse breeders’ associations. The endocrinological procedures of skeletal growth and development are controlled by hormones that are most likely to participate in ossification, such as insulin, thyroxin, growth hormone, parathyroid hormone, and calcitonin. Of the regulating proteins involved in animal ossification, matrilin 1 (MATN1), carboxypeptidase vitellog (CPVL), hyaluronan one (HYAL1), xin actin-binding repeat containing 2 (XIRP2), frizzled related protein (FRZB), collagens 5 and 9, (COL5A2, COL9A2), insulin-like growth factor (IGF1), and acyloxyacyl hydrolase (AOAH1) were mostly investigated in our earlier research [1,2] on the population of sport horses. Based on the literature, it can be assumed that the polymorphism of the genes encoding the proteins mentioned above was found to be associated with the occurrence of the size of different joints, being connected with developmental orthopedic diseases.

Two of the examined polymorphisms (in the *MATN1* and *CPVL* genes) were located in exons. The gene *MATN1* encodes the cartilage-specific cartilage matrix protein. Loughlin et al. [3] found that human linkage studies demonstrated that the *MATN1* gene segregated independently of several heritable chondrodysplasias, while Meulenbelt et al. [4] found a significant association between the *MATN1* gene and radiographically evident problems with bone formation. Another functional candidate gene for bone formation investigated is the *CPVL*. The *CPVL* gene had been examined previously [5] in osteochondrosis. But in the study of Wypchło et al. [2], one SNP (rs69569814) of the *CPVL* gene was found to be statistically significant in the determination of the occurrence of bone formation in fetlock joints after performance tests. The protein encoded by *CPVL* bears strong sequence similarity to serine carboxypeptidases. From the literature, it has been shown that *CPVL* expression is induced during monocyte maturation into macrophages. The *CPVL* gene, possibly connected with the immune response [6], as well as *MATN1*, are located in the QTL region strongly associated with bone formation. The mutations located within these genes are causal and cause a change in codon meaning, and it can affect the structure and function of the protein. Other polymorphisms investigated in earlier research were located in the regulatory areas: 5′UTR (*HYAL1*) and 3′UTR (*FRZB*), and three polymorphisms (in *XIRP2*, *COL5A2*, *IGF1*, and *AOAH* genes) were located in introns. FRZB is involved in skeleton development [7], and the *FRZB* gene, involved in human bone formation [8,9], is also expressed in equine cartilage. Simultaneously, hyaluronidases (HYALs) intracellularly degrade hyaluronan, which is one of the major glycosaminoglycans of the extracellular matrix. Hyaluronan is an important integral structural component of articular cartilage, acting as a lubricant in joints. Hyaluronan contributes to tissue hydrodynamics, proper movement, cell proliferation, migration, and differentiation. The XIRP2 family is a novel group of actin-binding proteins whose functions are not yet well understood [10], but polymorphism of the *COL5A2* gene, even though there is no causal mutation, appears to have an influence on symptoms of the bone disease. Collagen is a protein that strengthens many tissues, including cartilage, bones, skin, and sclera. The *COL5A1* gene codes for the main component of type V collagen. It is found in tissues containing type I collagen and regulates the structure of heterotypic fibers that consist of two types of collagen. The *COL9A2* gene encodes the alpha-2 subunit of collagen type IX. Type IX collagen, a heterotrimer of alpha-1 (COL9A1), alpha-2, and alpha-3 (COL9A2; COL9A3) chains specific to this type of collagen, is a cartilage-specific fibril-associated collagen. It is an important component of cartilage. The tough and flexible tissue constitutes much of the skeleton during early development. It is suggested that reduced chondrocyte differentiation, caused by lower plasma IGF-1 concentration, may contribute to the development of bone [11]. Finally, the *AOAH* gene encodes the hydrolase enzyme, which hydrolyses the secondary fatty acyl chains of bacterial polysaccharides, thus causing the detoxification of these molecules. The encoded protein may also play a role in the modulated host inflammatory response to Gram-negative bacteria. Three SNPs of the acyloxyacyl hydrolase (AOAH) gene were found to be significantly associated with bone formation in fetlock joints [5]. According to the literature, other genes whose polymorphism may be related to the skeleton, and whose metabolic pathways are involved in ossification processes, are as follows: Anillin actin-binding protein *ANLN*, BMP-binding endothelial regulator (*BMPER*), Transforming Growth Factor Beta (*TGF-β*), Engulfment and cell motility 1 (*ELMO1)*, and actin 1 receptor type I (ACVR). TGF-β is a polypeptide belonging to the transforming growth factor beta superfamily of cytokines. It is a secreted protein that performs many cellular functions, including the control of cell growth, cell proliferation, cell differentiation, and apoptosis. TGF-β plays an important role in growth cartilage metabolism, particularly in the control of chondrocyte differentiation and hypertrophy [12,13,14], also in bone remodeling. Semevolos et al. [15] found a higher (but not significant) expression of TGF-β in affected tissue. The *BMPR* gene was shown to be involved in BMP2- and BMP4-dependent osteoblast differentiation and BMP-dependent differentiation of the chondrogenic cells [16]. All the genes listed above may be potential markers of features related to the development of the skeletal system, and the studied polymorphism may be related to the varying intensity of skeletal changes.

## 2. Results

The obtained results confirm the hypothesis that the selected genes were closely associated with bone formation and size; however, their influence is not the same for all bone structures. Most genes were significant for the majority of the fetlock and hock measurements. For both joints, they affected from 1 (4%) to 14 (61%) of measurements performed, for single joints from 0 to 89% (Table 1, Table 2 and Table 3).

The most distinct effects in fetlock and hock structures were observed for *XIRP2, ELMO1*, when *ELMO1* was significant for all fetlock structures, but only for 1 out of 14 for measurement A—“the widest of the tibia epiphysis”. The *XIRP2* gene affected the measurements in the other way—no fetlock structures were influenced by this gene polymorphism, but two structures of the hock were affected: G—“length of the calcaneus” and N—“overall joint length”. In most cases, the number of measurements affected by the selected genes was higher for the fetlock measurements; however, it was not the case for *BMPER* and *XIRP2* (mentioned above), when the number of affected bone structures was higher for the hock joint.

For the fetlock, the most significant gene polymorphism was noted for the measurements of the bone width, depending on the localization of the measurement (epiphyses or middle of the long bones measurements A-B2). The bone cortical substance measurements and related measurements (C, C1, and C2) were less affected by selected genes, except for *COL9A2*, *AOAH1*, *CPVL*, *ACVR1*, and *IGF1* genes, which showed more cartilage-specific effects. The less affected measurement (D), being the size between the bones in the fetlock joint, is significantly affected by the polymorphisms of *CPVL*, *ACVR1*, and *HYAL3* genes, which are also functional cartilage genes. The length of the bone in the fetlock was not affected by only five genes: *COL9A2*, *FRZB*, *BMPER*, *XIRP2*, and *TGF-β*.

The hock measurements were not so specifically affected as the fetlock structures. Most structures were affected; however, by the *BMPER* and *ANLN* polymorphism of the genes. The highest number of measurements performed for the hock joint described the widths or thickness of different bone structures. The length bone structure measurements (D, G, and N) were influenced, except for genes mentioned above, the *COL5A2*, *FRZB*, and *CPVL*, but usually on a less significant level (*p* < 0.05).

The larger size of the bone structure was achieved in the fetlock for the heterozygotes of *COL9A2*, *AOAH1*, *BMPER*, *HYAL3*, and *ELMO1*. The heterozygotes were superior to homozygotes in the hock for the following genes: *COL9A2*, *HYAL3*, *ANLN*, and *HYAL1*. The lower homozygote values were obtained for GG in *CPVL* in fetlock measurements, TT for *HYAL3* in fetlock, TT for *ANLN* in fetlock, CC for *FRZB* in the hock, TT for *MATN* in the hock, and TT for *COL5A2* in the hock than their opposite homozygote and heterozygote variants. As observed, depending on the bone localization, *COL9A2* and *HYAL3* are expressed in the same way for most of the bone structures in both joints.

## 3. Discussion

The results showed that selected genes do not affect measured structures in the same way, likely due to the specificity of the analyzed bone structures. Even if cartilage samples from the examined joint were collected at a similar time, some may be slightly less mature, as the timing of cartilage development can vary between breeds. The measured bone structures are composed of distinct and specific tissue types, so genes primarily affecting cartilage may have less influence on overall bone measurements. Additional differences may also be observed because osteoblast–osteoclast ossification is a continuous process, so some differences could be captured in both measurements and gene expression. Some differences between the joints may also result from unequal numbers of observations. The amount of data for the fetlock joint appears sufficient, exceeding 1500 observations, while the hock measurements are less numerous. Nevertheless, the number of observations was enough for finding significant gene connections between polymorphism and measurements for *BMPER* and *ANLN* or *XIRP2* when such connections were not found for larger representatives of the fetlock measurements.

Another issue may be related to the specificity of the genes. As noted earlier in the introduction, some of them are associated with pathological conditions. Selected genes are involved in the process of bone formation at different stages, so the tissue formation can be dependent on immune response (IGF1) or by defects in bone formation (CPVL, FRZB). The *COL9A2* gene was found to be connected with musculoskeletal problems, as Collagens 9 is a family of proteins that support connective tissues, such as skin, bone, cartilage, tendons, and ligaments [17,18,19,20,21].

*FRZB* gene was found to be possibly breaking the cartilage integrity and connected with osteoarthritis [8,22,23,24]. *CPVL* gene is involved in macrophage production, also in bone disease inflammation, among others [25,26,27,28,29]. In horses, such studies are less evident. Different cartilage gene expressions were found in horses with osteochondral disease [30], which may also be relevant in our study, even if it was not directly investigated. RNA-sequencing revealed that biological pathways associated with skeletal morphogenesis were significantly enriched in horses affected by osteoporosis. The 30 differentially expressed genes in affected lymph nodes were associated with inflammatory responses [31].

According to Kemper et al. [32], skeletogenesis is complex and not yet fully understood. Derangement of this process likely underlies developmental skeletal pathologies. Approximately 1115 and 3574 genes were differentially expressed between age groups in adult articular cartilage and subchondral bone groups, respectively, with enriched pathways reflecting a state of growth, high metabolic activity, and tissue turnover.

Roberts et al. [33] note that the most recent data obtained from next-generation sequencing showed disturbances in the expression of genes for numerous proteoglycans and collagens. However, because of their low prevalence, not much progress has been made not only in improved diagnosis but also in our understanding of the biochemical basis and pathogenesis of these diseases in animals. This appears to be the case in our study, as well as different bone measurements that are not influenced by the same genes the same way.

Even after many years and numerous advancements, musculoskeletal injuries are still a welfare issue in sport and racehorses [34]. Data from multiple omics studies indicate that it is crucial for future research to address the effects of sample size, exercise, and normalization methods. We hope that our study will contribute to a broader understanding of these unresolved issues, as the dataset was extensive, and horses were kept under comparable conditions with the same training regime. Even if the amount of information between the fetlock and hock joints differs, it still provides valuable insights.

Because ossification processes are extremely important for horses, some studies aim to identify the genetic risk factors associated with bone diseases. The study by Palomino et al. [35] revealed 112 genes that were significantly differentially expressed in osteoblasts. Of these, forty-three genes have known roles in bone, 27 are not yet annotated in the equine genome, and 42 currently have no described role in bone. Another study by Stefaniuk-Szmukier M. et al. [36] investigated training-induced changes in transcript abundance of genes involved in the bone turnover process. Several potential candidate genes were identified and will be investigated in the future.

The evaluation of conformation traits is an important part of horse selection, as these traits are associated with performance, health, and longevity [37]. This study measured objectively joint angles in horses and, using Whole Genome Sequencing (WGS), identified three genes associated with measured joint angles—RSU1, PTER, and ALX1, along with several other suggestive QTLs. For our study, we have selected other genes. Additionally, we found no publications directly studying the association between horse bone measurements and gene polymorphism. The application of detailed measurements in genetic studies may improve our understanding of the underlying genetic effects of important traits in equine breeding.

Typically, when studying the association between genetic polymorphisms and animal phenotypes, selected animals should have no blood relationship within three generations to better represent a population. Such a selection of animals is very difficult in the Warmblood sport horse population, as they are genetically connected because of the wide use of the popular sires, which have high sport achievements. Connections between horses in our population were also observed, which could have influenced the results. However, such a collection of horses was necessary to maintain consistency for the environmental and training conditions. Another limitation of the study is the limited number of horses investigated. We have investigated all animals available over two years of the study from the training centers, and the number of measurements was high, particularly for the four fetlocks. The number of measurements for the hock was lower, although the number of parameters was higher than for the fetlock. This was due to the variable quality of the images, which in some cases made it difficult to accurately identify the necessary points, as well as the fact that the hock is a more complex joint than the fetlock. The conformation of the horse hock could influence the image quality. However, according to our knowledge, this study is the first one to study horse bone development measured objectively with a complex of genes involved in its formation. This novel study, even limited in the number of investigated horses, revealed a high number of significant associations. Overall, the genes affected 58% of the fetlock measurements and 31% of the hock measurements (Table 3). The HYAL 3 gene affected even 89% of fetlock parameters measured, and the BMPER gene 71% of the investigated parameters. Some of them were similar, but in total, the ANLN gene affected the highest number of measurements in both joints. These connections should be investigated further, especially since the direct association with one of the horse’s most common developmental orthopedic diseases is not particularly strong.

Investigations conducted on the same group of horses examined associations with osteochondrosis, which was evaluated subjectively by veterinarian specialists. The results showed a significant influence (*p* ≤ 0.05) of COL9A2 genotypes on the occurrence of osteochondrosis in both the fetlock and the hock joints. Although the polymorphism of this gene has not been proven to be a causal mutation, it appears to affect the symptoms of the disease. A second study confirmed seven SNPs located in the *MATN1*, *CPVL*, *HYAL1*, *XIRP2*, *FRZB*, *COL5A2*, and *IGF* genes were associated with the occurrence of osteochondrotic lesions in different joints (1,2). Osteochondrosis should probably be evaluated more objectively, if the associations between measurements and developmental diseases are stronger on the genetic level for objective traits than subjective veterinary evaluations. Even if the studied genes do not represent all problems with bone development, further studies should be conducted. A detailed, realistic, and country-independent evaluation of developmental bone diseases is of special importance for sport horse breeding.

Comparison of our results with the literature is challenging, as the genetic aspects of horse bone size are presented only at the general level, such as “body size”, “height at withers”, or “withers size”, which present exactly the same traits. Most such studies cite the *LCORL* and *NCAPG* genes as causative factors for the size of the horse [38,39,40,41,42,43,44,45,46,47,48,49]. This information, however, was not available during the planning of our research, and these genes were not considered informative for developmental orthopedic disorders. Other genes cited in the literature often are *LASP1* [41,42,43,44] and *ZFAT* [41,43]. In specific ponies, primitive horses, and donkey populations, several genes were mentioned as connected with the body size: *RFLNA*, *FOX01* [50], *TBX3* [51,52], *ANKRD1* [41], *GHR*, *SOX9*, *SOX11* [41], *NELL 1* [53], and *CYRIB* [54]. Some other genes were found significant for the equine morphology *SAMD7*, *SPARC1*, *IBSP*, *MEPE*, *DEUP12*, and *PLAAT1* [55] or body measurements *C4RORF33*, *CRB1*, *CPN1*, *FAM13A*, and *FGF12* [56]. Direct comparison between populations, even for the same traits, should be performed with caution, as there are some physiological differences in the genetic pathways underlying development across populations [57]. However, the mechanism of body growth is poorly understood [57], and studies that provide useful information for therapeutic strategies should be conducted. The energy metabolism found connected with *IGFBP-1* and *IGFBP-2* [43] genes seems physiologically connected with the significance of the *IGF1* gene for 67% of fetlock and 21% of the hock measured parameters in our study. Further studies on objective, measured traits should be provided to solve the issue of the unequal gene regulations for different kinds of the same bone measurements. As stated in our study, the same genes do not affect the bone width or length in the same way.

According to McIIwraith [58], prediction of musculoskeletal injury in the horse remains a challenge. The potential usefulness of various techniques to classify biomarkers at different stages of equine development was emphasized. The authors concluded that new information and studies on equine musculoskeletal biomarkers have potential translational value for humans and vice versa. Skeletal problems are equally important in humans and horses, and the welfare concerns associated with catastrophic musculoskeletal injury in horses further highlight the need for reliable and validated biomarkers in the horse.

## 4. Materials and Methods

### 4.1. Animals

Horses being trained in young horses’ performance tests for breeding, in total 198 were x-rayed twice in the beginning and at the end of the performance tests training (60 days for mares and 100 days for stallions). Performance tests for young horses were based on basic standardized training and conditioning at the official Polish Horse Breeders Association training stations. The performance tests for young horses take 100 days for stallions and 60 days for mares. During the performance tests, all horses are trained in riding and jumping, whereas stallions receive additional training in stamina skills. Training was conducted 6 days per week. The daily workload duration did not exceed 45 min. All veterinary interventions were conducted by a veterinary surgeon according to the veterinary procedures determined by the National Ethical Committee for Animal Experiments. Tested horses were aged 2.5 to 4.5 years. They were initially selected on the basis of their conformation, taking into account the average height at the withers (mean 165 cm; range 156–174 cm), the chest circumference (mean 190 cm; range 175–207) and the cannon bone circumference (mean 20.75 cm; range 18.5–22.5 cm) and evaluated for conformation (mean 78; range 75–84) in the scale up to 100 points. The pedigree analysis showed that the investigated horses were bred by 126 sires. There were 86 stallions and 112 mares. The collected group of animals was equal in conformation and consistent within pedigree. Horses were adults, and their fetlocks and hocks were mature.

### 4.2. Blood Samples and Gene Polymorphism

The gene polymorphism detection pipeline has been described previously in detail in our primary publications [1,2] and Supplementary Table S1, without any additional amplification reaction, as they were all preliminarily selected as connected with the bone development. Blood samples from all individuals were collected in 10 mL tubes containing potassium ethylenediaminetetraacetic acid (EDTA) as an anticoagulant and stored at −80 °C for DNA extraction. Acquisition of the blood was conducted by a veterinary surgeon according to the veterinary procedures. Extraction and purification of DNA were performed using the Wizard^®^ Genomic DNA Purification Kit (Promega, Pisz, Poland). DNA quality and purity were assessed using a NanoDrop 1000 Spectrophotometer, and stored at −20 °C. All SNPs in the candidate genes were analyzed using the same PCR-RFLP protocol. Primer pairs targeting the selected regions were designed with Primer3 and Primer-BLAST based on sequences obtained from GenBank. Amplification conditions were optimized individually for each primer pair and DNA fragment. Significant polymorphisms were identified in the following genes: *COL9A2*, *AOAH1*, *FRZB*, *CPVL*, *XIRP2*, *MATN1*, *ANLN*, *ACVR1*, *BMPER*, *HYAL3*, *HYAL1*, *TGF-β*, *IGF1*, *COL5A2*, and *ELMO1*. The basic polymorphism frequency of the investigated genes is presented in Table 4. The expected frequency, as well as the Hardy–Weinberg test, was performed. Five gene genotypes do not follow the Hardy–Weinberg equilibrium. The Hardy–Weinberg test was performed using the chi-square test.

### 4.3. Bone Structure Measurements

The X-rays were collected using Gierth 80 plus (Gierth SP.zoo, Wrocław, Poland) and digital scanner iCr3600 (MC-Imaging, LLC, Sperry, OK, USA) after sedation. The following joints were analyzed: metacarpophalangeal joints (front fetlocks), metatarsophalangeal joints (hind fetlocks), and tarsocrural (hock) joints. The lateromedial projection was taken into account for each joint. The measurements of the bone structure in both joints were performed using the program VetRay Vision 4.4.7 Vet Xp/2000. The characteristics of the joints were measured by 9 measurements for the fetlock joint and 14 measurements for the hock joint. The description of the measurements for both joints is presented in Table 5 and Table 6.

Because only images that provide the possibility to provide unquestionable measurements were taken into account, the number of observations is not always the same, and it is given in Table 7 and Table 8 with the basic statistical characteristics of the data.

All measurements described above were performed by experienced vets with radiological experience. Obtained measurements for the fetlock bone structures with basic statistical characteristics are presented in Table 7. The adequate measurements for the hock joint are presented in Table 8 with their basic characteristics.

### 4.4. Statistical Analysis

The analysis of variance (GLM, SAS 9.4 program) was used to explain the connections between gene polymorphism and the obtained measurements of the bone structures for the fetlock and hock joints. The data were evaluated together for the first and second stages of training. Additional effects that can influence the results were taken into account. The model of analysis included fixed effect of the following: sport horse breed register (Wielkopolski—52 horses, Małopolski—73, Polish Halfbred—50, and the group of foreign sport Warmbloods—23), training center (two places—83 and 115 horses each), gender (112 mares and 86 stallions), limb characteristics (left/right side, horse front/hind for the fetlock, before/after training) and gene polymorphisms. The limb characteristics were divided 50/50% for the measurements, so 1503–1559 observations were for the fetlock, and 149–188, depending on the bone structure. The least-squares means were evaluated based on the post hoc test. The formula of the statistical model was as follows:

**y_ijklmnop_ = µ + T_i_ + R_j_ + G_k_ + L_l_ + S_m_ + I_n_ + P_o_ + e_ijklmnop_**

where

**y_ijklmnop_**—bone measurement.
**µ**—mean.
**T_i_**—fixed effect of the training center (i = 1, 2).
**R_j_**—fixed effect of the warmblood register (j = 1, …, 4).
**G_k_**—fixed effect of the horse gender (k = 1, 2).
**L_l_**—fixed effect of the limb—front/hind (for fetlock joints k = 1, 2).
**S_m_**—fixed effect of the horse side (k = 1, 2).
**I_n_**–fixed effect of the investigations (k = 1, 2).
**P_o_**–fixed effect of the gene polymorphism (l = 1, 2, or 1, 2, 3).
**e_ijklmnop_**—error.

## Figures and Tables

**Table 1:** The effects of selected genes on the fetlock measurements.

GENES/ Genotypes		Measurements of the Fetlock Joint [LSM in cm]								
		A	B	B1	B2	C	C1	C2	D	E
*COL9A2*	CC	3.69 ^AB^	4.86 ^AB^	3.15 ^AB^	3.29 ^AB^	0.80 ^AB^	0.65 ^AB^	1.70	0.14	10.21
	CT	3.80 ^A^	4.94 ^A^	3.24 ^Ac^	3.35 ^A^	0.83 ^A^	0.68 ^Ac^	1.72	0.14	10.22
	TT	3.78 ^B^	4.92 ^B^	3.21 ^Bc^	3.25 ^B^	0.83 ^B^	0.67 ^Bc^	1.71	0.14	10.19
*p*-value		0.0001	0.01	0.001	0.0001	0.001	0.0001	ns	ns	ns
*AOAH1*	AA	3.74 ^AB^	4.90 ^AB^	3.19	3.33 ^a^	0.82	0.66 ^AB^	1,71 ^A^	0.14	10.19 ^a^
	AG	3.83 ^BC^	4.95 ^A^	3.21	3.36 ^ab^	0.83	0.69 ^BC^	1.71 ^B^	0.14	10.24 ^b^
	CG	3.54 ^AC^	4.81 ^B^	3.21	3.24 ^b^	0.77	0.57 ^AC^	1.88 ^AB^	0.14	10.54 ^ab^
*p*-value		0.0001	0.01	ns	0.01	0.07	0.0001	0.02	ns	0.02
*FRZB*	CC	3.68 ^aB^	4.84 ^aB^	3.17	3.29 ^ab^	0.82	0.67	1.67 ^a^	0.14	10.25
	CT	3.74 ^aC^	4.91 ^a^	3.20	3.33 ^a^	0.82	0.66 ^A^	1.72 ^ab^	0.14	10.20
	TT	3.78 ^BC^	4.92 ^B^	3.20	3.45 ^b^	0.82	0.68 ^A^	1.70 ^b^	0.14	10.21
*p*-value		0.0001	0.03	ns	0.04	ns	0.001	0.03	ns	ns
*CPVL*	CC	3.77	4.95 ^A^	3.19	3.35	0.83 ^AB^	0.68	1.69 ^ab^	0.14	10.28 ^AB^
	CG	3.75	4.89 ^Ab^	3.20	3.33	0.82 ^A^	0.67	1.71 ^a^	0.15 ^A^	10.19 ^A^
	GG	3.77	4.92 ^b^	3.19	3.33	0.81 ^B^	0.66	1.71 ^b^	0.13 ^A^	10.19 ^B^
*p*-value		ns	0.0007	ns	ns	0.0007	0.09	0.05	0.0001	0.004
*MATN*	CC	3.76	4.91	3.24 ^Ab^	3.27	0.81	0.70 ^AB^	1.72	0.15	10.24
	CT	3.76	4.90	3.19 ^Ac^	2.23	0.83	0.66 ^A^	1.71	0.15	10.19
	TT	3.76	4.91	3.21 ^bc^	3.34	0.82	0.67 ^B^	1.71	0.15	10.21
*p*-value		ns	ns	0.005	ns	ns	0.0003	ns	ns	ns
*ACVR1*	AA	3.76	4.95	3.19	3.39 ^a^	0.85 ^Ab^	0.67	1.68 ^a^	0.14	10.39 ^aB^
	TA	3.74	4.92	3.21	3.32 ^ab^	0.82 ^Ac^	0.65 ^A^	1.74 ^aB^	0.14 ^A^	10.25 ^aC^
	TT	3.77	4.91	3.20	3.43 ^b^	0.83 ^bc^	0.68 ^A^	1.70 ^B^	0.15 ^A^	10.19 ^BC^
*p*-value		ns	ns	ns	ns	0.009	0.0001	0.0001	0.003	0.002
*BMPER*	CC	3.77	4.88	3.06 ^AB^	3.33	0.80	0.66	1.61 ^AB^	0.15	10.33
	CT	3.75	4.87 ^A^	3.22 ^A^	3.27	0.82	0.67	1.72 ^A^	0.15	10.18
	TT	3.76	4.92 ^A^	3.18 ^B^	3.34	0.83	0.67	1.70 ^B^	0.15	10.21
*p*-value		ns	0.009	0.0001	ns	ns	ns	0.006	ns	ns
*HYAL3*	CC	3.78 ^a^	4.95 ^A^	3.21 ^a^	3.36 ^A^	0.82 ^a^	0.66	1.73 ^a^	0.14 ^A^	10.19 ^a^
	TC	3.79 ^B^	4.92 ^b^	3.22 ^B^	3.36 ^B^	0.83 ^aB^	0.67	1.72 ^b^	0.15 ^AB^	10.26 ^aB^
	TT	3.74 ^aB^	4.89 ^Ab^	3.18 ^aB^	3.31 ^AB^	0.82 ^B^	0.68	1.70 ^ab^	0.14 ^B^	10.17 ^B^
*p*-value		0.009	0.01	0.0001	0.001	0.01	ns	0.02	0.0005	0.0001
*IGF1*	AA	3.76	4.91 ^a^	3.20	3.34 ^a^	0.83 ^a^	0.67 ^A^	1.71 ^A^	0.15	10.20 ^a^
	AT	3.76	4.88 ^a^	3.20	3.32 ^a^	0.81 ^a^	0.65 ^A^	1.74 ^A^	0.14	10.27 ^a^
	TT	-	-	-	-	-	-	-	-	-
*p*-value		ns	0.04	ns	0.03	0.01	0.0001	0.008	ns	0.01
*XIRP2*	AA	3.76	4.89	3.19	3.32	0.83	0.67	1.69	0.15	10.19
	AG	3.76	4.92	3.20	3.34	0.82	0.67	1.72	0.15	10.22
	GG	3.76	4.89	3.19	3.34	0.82	0.67	1.71	0.15	10.20
*p*-value		ns	ns	ns	ns	ns	ns	ns	ns	ns
*ANLN*	CC	3.75 ^a^	4.93 ^a^	3.22 ^A^	3.34 ^A^	0.84 ^AB^	0.67	1.71	0.15	10.24 ^A^
	CT	3.78 ^aB^	4.91 ^b^	3.20	3.35 ^B^	0.82 ^A^	0.67	1.72	0.15	10.23 ^B^
	TT	3.73 ^B^	4.89 ^ab^	3.19 ^A^	3.30 ^AB^	0.82 ^B^	0.66	1.71	0.15	10.11 ^AB^
*p*-value		0.001	0.05	0.01	0.0001	0.0009	ns	ns	ns	0.0001
*HYAL1*	AA	3.77 ^A^	4.82 ^aB^	3.21	3.35 ^A^	0.83 ^a^	0.67	1.71	0.14	10.23 ^a^
	AC	3.70 ^A^	4.87 ^a^	3.19	3.29 ^A^	0.82 ^a^	0.66	1.71	0.14	10.18
	CC	3.74	4.83 ^B^	3.20	3.31	0.81	0.69	1.75	0.14	10.10 ^a^
*p*-value		0.0001	0.002	ns	0.0001	0.02	0.09	ns	ns	0.02
*TGF-β*	GG	3.74 ^A^	4.88 ^aB^	3.18 ^A^	3.32 ^A^	0.82	0.66 ^aB^	1.71	0.15	10.19
	TG	3.76 ^b^	4.91 ^aC^	3.20 ^b^	3.34 ^B^	0.82	0.67 ^a^	1.71	0.15	10.23
	TT	3.79 ^Ab^	4.98 ^BC^	3.23 ^Ab^	3.37 ^AB^	0.83	0.68 ^B^	1.73	0.14	10.20
*p*-value		0.007	0.0001	0.0009	0.0003	ns	0.01	ns	ns	ns
*COL5A2*	CC	3.84 ^AB^	4.97 ^AB^	3.23 ^A^	3.35	0.83 ^a^	0.68	1.73 ^a^	0.15 ^Ab^	10.24
	CT	3.75 ^A^	4.91 ^A^	3.17 ^AB^	3.35	0.82 ^aB^	0.67	1.69 ^ab^	0.13 ^A^	10.26 ^A^
	TT	3.75 ^B^	4.90 ^B^	3.21 ^B^	3.33	0.83 ^B^	0.67	1.72 ^b^	0.14 ^b^	10.18 ^A^
*p*-value		0.0001	0.0034	0.0001	ns	0.01	ns	0.02	0.02	0.01
*ELMO1*	GG	3.75 ^A^	4.90 ^a^	3.18 ^A^	3.32 ^A^	0.82	0.66 ^A^	1.70 ^A^	0.14	10.17 ^A^
	TG	3.79 ^AB^	4.92 ^aB^	3.23 ^AB^	3.36 ^AB^	0.83	0.68 ^AB^	1.73 ^A^	0.15	10.27 ^AB^
	TT	3.73 ^B^	4.88 ^B^	3.19 ^B^	3.33 ^B^	0.82	0.66 ^B^	1.72	0.15	10.18 ^B^
*p*-value		0.0001	0.0004	0.0001	0.002	0.08	0.001	0.01	0.08	0.0001

Statistically significant differences marked “A” for *p* < 0.01 or “a” for *p* < 0.05; “B” for *p* < 0.01 or “b” for *p* < 0.05; “C” for *p* < 0.01 or “c” for *p* < 0.05, ns—non-significant.

**Table 2:** The effects of selected genes on the hock measurements.

GENES/ Genotypes		Measurements of the Hock Joint [LSM in cm]													
		A	B	C	D	E	F	G	H	I	J	K	L	M	N
*COL9A2*	CC	7.83	1.45 ^A^	1.58	3.31	5.14	6.51 ^a^	11.95	7.23 ^aC^	5.94	1.12	1.63 ^ab^	1.61	6.36	17.80 ^a^
	CT	7.72	1.55 ^B^	1.57	3.38	5.15	6.56 ^B^	12.06	7.39 ^bC^	6.01	1.18	1.68 ^a^	1.65	6.57	18.14 ^a^
	TT	7.63	1.47 ^AB^	1.63	3.65	5.04	6.28 ^aB^	11.94	7.36 ^ab^	5.93	1.13	1.67 ^b^	1.62	6.56	17.94
*p*-value		ns	0.001	ns	ns	ns	0.01	ns	0.05	ns	0.09	0.04	ns	ns	0.03
*AOAH1*	AA	7.74	1.53 ^A^	1.57 ^A^	3.38	5.10	6.42	12.00	7.29	5.88	1.19	1.68	1.65	6.51	17.95
	AG	7.66	1.45 ^A^	1.65 ^A^	3.31	5.12	6.52	11.97	7.45	5.93	1.08	1.64	1.60	6.51	18.12
	CG	-	-	-	-	-	-	-	-	-	-	-	-	-	-
*p*-value		ns	0.005	0.05	ns	ns	ns	ns	0.07	ns	0.002	ns	0.07	ns	ns
*FRZB*	CC	7.07 ^Ab^	1.52	1.51	3.26	4.84	6.12	11.52 ^AB^	6.97 ^AB^	5.58 ^AB^	1.18	1.51	1.67	6.15	17.36 ^AB^
	CT	7.92 ^AC^	1.52	1.58	3.37	5.14	6.45	12.01 ^A^	7.39 ^A^	6.03 ^A^	1.16	1.68	1.65	6.63	18.03 ^A^
	TT	7.66 ^bC^	1.49	1.61	3.36	5.14	6.50	12.06 ^B^	7.36 ^B^	5.99 ^B^	1.14	1.67	1.61	6.47	18.06 ^B^
*p*-value		0.006	ns	ns	ns	ns	ns	0.01	0.01	0.002	ns	ns	ns	ns	0.01
*CPVL*	CC	7.61	1.35 ^AB^	1.60	3.29 ^aB^	4.97 ^A^	6.39	11.99	7.33	5.94	1.15	1.64	1.58 ^A^	6.36	18.09 ^a^
	CG	7.72	1.52 ^A^	1.62	3.37 ^a^	5.19 ^Ab^	6.56 ^A^	12.04	7.39	6.00	1.15	1.66	1.66 ^A^	6.49	18.07 ^b^
	GG	7.72	1.51 ^B^	1.57	3.3 ^B^	5.07 ^b^	6.36 ^A^	11.94	7.29	5.93	1.17	1.69	1.62	6.63	17.81 ^ab^
*p*-value		ns	ns	ns	0.02	0.005	0.05	ns	ns	ns	ns	ns	0.009	ns	0.03
*MATN*	CC	7.61	1.35 ^AB^	1.58	3.57 ^ab^	5.16	6.29	11.92	7.65	5.96	1.30 ^a^	1.73	1.83	6.80	17.96
	CT	7.72	1.52 ^A^	1.61	3.38 ^a^	5.09	6.48	11.87 ^a^	7.33	5.95	1.18	1.68	1.63	6.46	17.90
	TT	7,72	1.51 ^B^	1.59	3.34 ^b^	5.12	6.44	12.06 ^a^	7.33	5.98	1.14 ^a^	1.66	1.63	6.53	18.05
*p*-value		ns	0.003	ns	0.05	ns	0.005	ns	0.05	ns	ns	0.04	0.007	ns	ns
*ACVR1*	AA	-	-	-	-	-	-	-	-	-	-	-	-	-	-
	TA	7.72	1.49	1.61	3.31 ^a^	4.96 ^A^	6.40	11.96	7.26 ^a^	5.96	1.18	1.64	1.62	6.41	17.93
	TT	7.72	1.52	1.59	3.38 ^a^	5.16 ^A^	6.48	12.01	7.37 ^a^	5.97	1.15	1.68	1.64	6.56	18.03
*p*-value		ns	ns	ns	0.03	0.001	ns	ns	0.05	ns	ns	ns	ns	ns	ns
*BMPER*	CC	8.57 ^a^	1.55	1.62	3.33	5.56 ^ab^	6.85	12.41 ^a^	7.57 ^a^	6.38 ^Ab^	0.99 ^a^	1.63	1.61	6.48	18.46
	CT	7.51 ^a^	1.46	1.52	3.17 ^A^	5.09 ^a^	6.63	11.75 ^ab^	7.13 ^aB^	5.78 ^AC^	1.07 ^B^	1.59 ^a^	1.53 ^A^	6.44	17.74
	TT	7.74	1.51	1.60	3.37 ^A^	5.10 ^b^	6.43	12.01 ^b^	7.36 ^B^	5.98 ^bC^	1.17 ^aB^	1.68 ^a^	1.65 ^A^	6.52	18.02
*p*-value		0.05	0.07	ns	0.001	0.04	ns	0.01	0.007	ns	0.002	0.03	0.005	ns	0.06
*HYAL3*	CC	7.77	1.51	1.63	3.31	4.91 ^A^	6.56	11.94	7.30	5.94	1.19	1.63 ^a^	1.61	6.74	17.98
	TC	7.72	1.51	1.60	3.38	5.29 ^Ab^	6.38	12.08 ^A^	7.37	5.97	1.15	1.69 ^aB^	1.64	6.50	18.03
	TT	7.68	1.51	1.58	3.34	5.05 ^b^	6.54	11.86 ^A^	7.31	5.98	1.15	1.63 ^B^	1.63	6.44	17.95
*p*-value		ns	ns	ns	ns	0.005	ns	0.01	ns	ns	ns	0.009	ns	ns	ns
*IGF1*	AA	7.73	1.51 ^a^	1.60	3.36	5.10 ^a^	6.46	11.99	7.34	5.96	1.16	1.66	1.64 ^A^	6.52	18.00
	AT	7.62	1.45 ^a^	1.59	3.36	5.28 ^a^	6.30	12.10	7.47	6.07	1.13	1.69	1.56 ^A^	6.46	17.96
	TT	-	-	-	-	-	-	-	-	-	-	-	-	-	-
*p*-value		ns	0.01	ns	ns	0.03	ns	ns	ns	ns	ns	ns	0.002	ns	ns
*XIRP2*	AA	7.71	1.51	1.61	3.35	5.14	6.39	11.95 ^a^	7.32	5.94	1.13	1.67	1.66	6.39	17.89
	AG	7.68	1.51	1.60	3.36	5.08	6.44	11.95 ^B^	7.32	5.95	1.16	1.66	1.63	6.47	17.97
	GG	7.82	1.51	1.60	3.36	5.19	6.57	12.20 ^aB^	7.44	6.06	1.15	1.68	1.63	6.66	18.18
*p*-value		ns	ns	ns	ns	ns	ns	0.01	ns	ns	ns	ns	ns	ns	0.07
*ANLN*	CC	7.68	1.53 ^A^	1.59	3.34 ^a^	5.01 ^A^	6.46	11.86 ^A^	7.26 ^a^	5.93	1.17	1.63 ^A^	1.63	6.44	17.86 ^A^
	CT	7.78	1.52 ^B^	1.61	3.38 ^B^	5.18 ^A^	6.45	12.11 ^AB^	7.42 ^ab^	6.01 ^A^	1.16	1.69 ^AB^	1.64	6.53	18.18 ^AB^
	TT	7.51	1.43 ^AB^	1.56	3.26 ^aB^	5.09	6.43	11.78 ^B^	7.28 ^b^	5.86 ^A^	1.12	1.61 ^B^	1.62	6.66	17.63 ^B^
*p*-value		ns	0.001	ns	0.004	0.02	ns	0.004	0.02	0.01	ns	0.002	ns	ns	0.001
*HYAL1*	AA	7.92 ^A^	1.51	1.59	3.37	5.11	6.43	12.08 ^a^	7.29	5.97	1.13	1.70	1.62	6.50	18.05
	AC	7.98 ^B^	1.53	1.63	3.38	5.21	6.53	12.28 ^ab^	7.25	6.04	1.14	1.68	1.62	6.81	18.20
	CC	6.84 ^AB^	1.52	1.57	3.34	5.08	6.50	11.70 ^b^	7.60	5.94	1.23	1.57	1.71	6.43	17.79
*p*-value		0.002	ns	ns	ns	ns	ns	0.03	ns	ns	ns	ns	ns	ns	ns
*TGF-β*	GG	7.82	1.52	1.59	3.37	5.15	6.35	12.04	7.29	5.97	1.16 ^A^	1.68	1.66 ^a^	6.69	18.09
	TG	7.65	1.50	1.60	3.34	5.10	6.48	11.95	7.34	5.95	1.13 ^B^	1.66	1.61 ^ab^	6.43	17.94
	TT	7.77	1.54	1.60	3.42	5.11	6.54	12.04	7.45	6.07	1.26 ^AB^	1.68	1.66 ^b^	6.50	18.12
*p*-value		ns	ns	ns	ns	ns	ns	ns	ns	ns	0.001	ns	0.03	ns	ns
*COL5A9*	CC	7.91	1.52	1.61	3.45 ^ab^	5.00 ^A^	6.45	12.17	7.47	6.22 ^AB^	1.18 ^a^	1.69	1.66	6.48	18.36 ^aB^
	CT	7.85 ^a^	1.51	1.56	3.34 ^a^	5.26 ^AB^	6.45	11.96	7.34	5.94 ^A^	1.09 ^aB^	1.69	1.61	6.69	17.98 ^a^
	TT	7.62 ^a^	1.51	1.61	3.34 ^b^	5.09 ^B^	6.45	11.97	7.31	5.94 ^B^	1.17 ^B^	1.66	1.63	6.47	17.94 ^B^
*p*-value		0.05	ns	ns	0.03	0.004	ns	ns	ns	0.003	0.003	ns	ns	ns	0.01
*ELMO1*	GG	7.72	1.49	1.61	3.37	5.15	6.43	12.06	7.32	5.95	1.16	1.68	1.64	6.44	17.95
	TG	7.71	1.52	1.57	3.36	5.11	6.51	12.00	7.39	5.99	1.17	1.67	1.62	6.65	18.06
	TT	7.72	1.53	1.61	3.35	5.06	6.42	11.92	7.31	5.96	1.13	1.65	1.65	6.42	17.98
*p*-value		ns	0.08	ns	ns	ns	ns	ns	ns	ns	ns	ns	ns	ns	ns

Statistically significant differences marked “A” for *p* < 0.01 or “a” for *p* < 0.05; “B” for *p* < 0.01 or “b” for *p* < 0.05; “C” for *p* < 0.01 or “c” for *p* < 0.05, ns—non-significant.

**Table 3:** The summary of the effects of selected genes on measurements.

GENES	Number of Affected Measurements					
	Fetlock (9 Parameters)		Hock (14 Parameters)		Both Joints (23 Parameters)	
	Number Affected	% Affected	Number Affected	% Affected	Number Affected	% Affected
*COL9A2*	6	67	6	43	12	52
*AOAH1*	7	78	5	36	12	52
*FRZB*	5	56	5	36	10	43
*CPVL*	6	67	5	36	11	48
*MATN*	2	22	6	43	8	35
*ACVR1*	5	56	3	21	8	35
*BMPER*	3	33	10	71	13	56
*HYAL3*	8	89	3	21	11	48
*IGF1*	6	67	3	21	9	39
*XIRP2*	0	0	1	7	1	4
*ANLN*	6	67	8	57	14	61
*HYAL1*	5	56	2	14	7	30
*TGF-β*	5	56	2	14	7	30
*COL5A2*	7	78	6	43	13	57
*ELMO1*	7	78	0	0	7	30
mean	5	58	4	31	10	41

**Table 4:** The genes genotypes, allele frequencies, and Hardy–Weinberg test results.

GENES		Gene Genotype	Gene Frequency		Allele Frequency Observed	
			Observed	Expected		
*COL9A2* (n = 198)	CC	61	0.310	0.200	C	0.447
	CT	54	0.274	0.494	-	
	TT	83	0.416	0.306	T	0.553
Hardy–Weinberg test			Χ^2^ = 39.09	*p* = 0.0001		
*AOAH1* (n = 198)	AA	168	0.848	0.850	A	0.922
	AG	29	0.146	0.144	-	
	GG	1	0.0005	0.0006	G	0.078
Hardy–Weinberg test			Χ^2^ = 0.04	*p* = 0.83354		
*FRZB* (n = 198)	CC	8	0.004	0.064	C	0.253
	CT	84	0.424	0.378	-	
	TT	106	0.535	0.559	T	0.747
Hardy–Weinberg test			Χ^2^ = 3.03	*p* = 0.08154		
*CPVL* (n = 198)	CC	38	0.192	0.189	C	0.434
	CG	96	0.485	0.491	-	
	GG	64	0.323	0.320	G	0.566
Hardy–Weinberg test			Χ^2^ = 0.03	*p* = 0.85167		
*MATN* (n = 198)	CC	9	0.045	0.046	C	0.215
	CT	67	0.338	0.337	-	
	TT	122	0.616	0.617	T	0.785
Hardy–Weinberg test			Χ^2^ = 0.00	*p* = 0.95882		
*ACVR1* (n = 198)	AA	5	0.025	0.025	A	0.157
	TA	52	0.263	0.264	-	
	TT	141	0.712	0.711	T	0.843
Hardy–Weinberg test			Χ^2^ = 0.01	*p* = 0.93717		
*BMPER* (n = 198)	CC	3	0.015	0.0009	C	0.096
	CT	32	0.162	0.174	-	
	TT	163	0.823	0.817	T	0.904
Hardy–Weinberg test			Χ^2^ = 0.93	*p* = 0.03353		
*HYAL3* (n = 198)	CC	25	0.126	0.132	C	0.364
	TC	94	0.475	0.463	-	
	TT	79	0.399	0.405	T	0.636
Hardy–Weinberg test			Χ^2^ = 0.13	*p* = 0.71664		
*IGF1* (n = 198)	AA	168	0.848	0.854	A	0.924
	AT	30	0.152	0.140	-	
	TT	-	0.000	0.006	T	0.076
Hardy–Weinberg test			Χ^2^ = 1.33	*p* = 0.24881		
*XIRP2* (n = 198)	AA	43	0.217	0.223	A	0.472
	AG	101	0.510	0.498	-	
	GG	54	0.273	0.279	G	0.528
Hardy–Weinberg test			Χ^2^ = 0.11	*p* = 0.74237		
*ANLN* (n = 198)	CC	58	0.293	0.271	C	0.520
	CT	90	0.455	0.499	-	
	TT	50	0.253	0.230	T	0.480
Hardy–Weinberg test			Χ^2^ = 1.58	*p* = 0.20829		
*HYAL1* (n = 198)	AA	158	0.798	0.781	A	0.884
	AC	34	0.172	0.205	-	
	CC	6	0.030	0.013	C	0.116
Hardy–Weinberg test			Χ^2^ = 5.31	*p* = 0.02120		
*TGF-β* (n = 198)	GG	68	0.343	0.343	G	0.586
	TG	96	0.485	0.485	-	
	TT	34	0.172	0.172	T	0.414
Hardy–Weinberg test			Χ^2^ = 0.00	*p* = 0.99056		
*COL5A9* (n = 198)	CC	17	0.086	0.051	C	0.225
	CT	55	0.278	0.348	-	
	TT	126	0.0636	0.601	T	0.775
Hardy–Weinberg test			Χ^2^= 8.15	*p* = 0.00431		
*ELMO1* (n = 198)	GG	80	0.404	0.361	G	0.601
	TG	78	0.394	0.480	-	
	TT	40	0.202	0.159	T	0.399
Hardy–Weinberg test			Χ^2^ = 6.32	*p* = 0.01197		

**Table 5:** Description of measurements of each hock joint (Supplementary Materials Figures S1–S6).

Measurement	Description
A	The line marked with the letter A passes through the widest part of the distal (inferior) epiphyses of the tibia, and it is perpendicular to the long axis of this bone
B	The line marked with the letter B runs on the surface of the central tarsal bone from the point where the fissure of proximal intertarsal joint begins to the point where the fissure of distal intertarsal joint begins
C	The line marked with the letter C runs on the surface of the third tarsal bone from the point where the fissure of distal intertarsal joint begins to the point where the fissure of tarsometatarsal joint begins
D	Line D runs as an extension of the long axis of the 3rd metatarsal bone from the point on the central tarsal bone to the point on the 3rd tarsal bone.
E	The line marked with the letter E connects two the most external and the lowest points of the tibial cochlea
F	The line marked with the letter F extends from the end of the joint gap limited by the coronoid process to the end of talocalcaneal join between the calcaneus and the talus
G	The line marked with the letter G extends from the point in the middle of the F line to the highest point on the calcaneal tuberosity of the calcaneus
H	The line marked with letter H occurs at the widest part of the calcaneal tuberosity of the calcaneus from the outermost point of the dorsal side of this tuberosity to the outermost point of the plantar side of this tuberosity
I	The line marked with the letter I runs perpendicular to the G line in the middle of the G line
J	The J line represents the thickness of the dorsal cortical bone of the tibia at the exact distance which is the value of the D line drawn along the long axis of the tibia from the beginning of proximal epiphysis of the tibia. The J line runs perpendicular to the long axis of the tibia
K	The K line marks the thickness of the central tarsal bone along the long axis of the 3rd metatarsal bone
L	The L line marks the thickness of the third tarsal bone along the long axis of the 3rd metatarsal bone
M	The line marked with the letter M runs at the widest part of the proximal epiphysis of the 3rd metatarsal bone and it is perpendicular to the long axis of this bone
N	The line marked with the letter N runs from the lowest point of the sagittal sulcus within the calcaneal tuberosity of the calcaneus to the starting point of the tarsometatarsal joint on the 3rd metatarsal bone

**Table 6:** Description of nine measurements of each fetlock joint (Supplementary Materials Figures S7–S15).

Measurement	Description
A	Dimension A runs from the beginning of the median crest of the distal metacarpal/metatarsal bone III to the beginning of the epicondyle
B	Dimension B includes the width of the proximal fetlock bone and runs parallel to the line connecting the two highest visible points of the joint surface of the fetlock joint, at its widest point
B1	The dimension marked with the letter B1 runs halfway along the fetlock bone, perpendicular to the long axis of this bone, from the upper border of the dorsal cortex to the lower border of the ventral cortex
B2	The dimension marked with the letter B2 is drawn parallel to the line connecting the two highest visible points of the proximal fetlock bone at the widest point of the distal fetlock bone
C	The dimension marked with the letter C determines the thickness of the dorsal cortex of the fetlock bone at the mid-length of the bone.
C1	The dimension marked with the letter C1 determines the thickness of the cortical substance of the ventral surface of the fetlock bone at the mid-length of the bone
C2	The dimension marked with the letter C2 denotes the distance between the inner borders of the dorsal and ventral cortex at the midpoint of the fetlock bone.
D	The dimension marked with the letter D indicates the width of the fetlock joint chink at its lowest point, measured from the surface of the distal metacarpal/metatarsal III bone to the surface of the proximal epiphysis of the fetlock bone. This section is aligned with the long axis of the fetlock bone.
E	The dimension marked with the letter E indicates the length of the fetlock bone from the lowest point of the proximal epiphysis in the fetlock joint to the lowest point of the distal epiphysis of the fetlock bone in the coronal joint

**Table 7:** Basic measurement characteristics of the bone structures for the fetlock joints.

Measurement (cm)		Mean	SD	Coefficient of Variability
A	N = 1559	3.74	0.23	6.15
B	N = 1559	4.88	0.27	5.53
B1	N = 1559	3.17	0.17	5.36
B2	N = 1559	3.31	0.18	5.44
C	N = 1559	0.82	0.08	9.76
C1	N = 1559	0.66	0.08	12.12
C2	N = 1503	1.68	0.19	11.31
D	N = 1558	0.14	0.03	21.43
E	N = 1559	10.13	0.46	4.54

**Table 8:** Basic measurement characteristics of the bone structures for the hock joints.

Measurement (cm)		Mean	SD	Coefficient of Variability
A	N = 181	7.76	0.70	9.02
B	N = 188	1.48	0.12	8.11
C	N = 191	1.58	0.18	11.39
D	N = 181	2.28	0.23	10.09
E	N = 177	5.05	0.37	7.33
F	N = 174	6.33	0.53	8.37
G	N = 149	11.79	0.56	4.75
H	N = 172	7.32	0.38	5.19
I	N = 149	5.87	0.31	5.28
J	N = 172	1.11	0.17	15.32
K	N = 173	1.62	0.15	9.26
L	N = 173	1.59	0.12	7.55
M	N = 178	6.47	0.96	14.84
N	N = 169	17.71	0.83	4.69

## Data Availability

Data can be available from the corresponding author upon reasonable request.

## Supplementary Materials

The following supporting information can be downloaded at: https://www.mdpi.com/article/10.3390/ijms26199645/s1. References [1,2,5,59,60,61,62,63] are cited in the Supplementary Materials.
