# Supplementary material for: Connections Between Gene Polymorphism and Fetlock and Hock Measurements in Polish Sport Horses
Source: Int J Mol Sci. 2025 Oct 2;26(19):9645. doi: 10.3390/ijms26199645 (PMC12525504; doi:10.3390/ijms26199645)
Supplement: Supplementary file 1 [file ijms-26-09645-s001.zip › ijms-3861614-Table S1.pdf]

Supplementary Table S1. Summary of the gene characteristics and methodology.

Gene	Enzyme	Fragment size (pz)	Change	ECA	SNP/place	Methods
COL9A2	BstUI	C:105<br>T:490	Exon 12, T/C	ECA2	Rs397140642<br>/17803980	Komm 2010
AOHA1	CviKI-1	A:83<br>G:76	Exon 21, A/G	ECA4	AJ543065:g.703	Wypchło et al.2018
FRZB	HpyCH4IV	C:31 64, 96 i 250<br>T:31,160,250	Exon 4, C/T	ECA18	Rs393854394/<br>60407729	Wittwer 2006;<br>Wypchło et al.2017
*ACVR*1	BccI	T: 45, 176, 368<br>A: 221, 368	Exon 10, T/A	ECA18	Rs393965603/<br>38688224	Wittwer 2006
ANLN	AFlIII	C:124,<br>T: 46,78	Exon 4, C/T	ECA4	Rs69538671/<br>65879635	Wittwer et al. 2008,
CPVL	BstNI	C: 56, 67;<br>G: 123	Exon 11, G/C	ECA4	Rs69569814/<br>59894762	Wypchło et al.2018
*XIRP*2	BsaWI	G: 326, 481;<br>A: 807	Intron, A/G	ECA18	AJ885515:g.159/<br>46892864	Wittwer et al.2009
*MATN*1	Mn1I	C:39,185,246<br>T:185,285	Exon 5, C/T	ECA2	Rs396491703/<br>25766179	Dierks 2006
BMPER	HphI	C:208<br>T:83,125	Intron T/C	ECA4	1236768681:g.188/<br>63992987	Wittwer et al.2008,
ELMO1	MboII	T:42,54,69,76<br>G: 54,69,118	5'UTR, G/T	ECA4	Rs782828177/<br>66283446	Wittwer et al.2008
HYAL1	NlaII	C:73<br>A:21,52	5'UTR, A/C	ECA16	Rs397148579/<br>3694833	Wypchło et al.2018
HYAL3	BtgI	C:15, 24, 66<br>T: 24,81	Ekson2, C/T	ECA16	Rs69233290/<br>36964482	Lampe 2009
COL5A2	MluCI	C:11,133<br>T:11,36,97	Intron, C/T	ECA18	Rs69137085/<br>65557759	Dierks 2006
IGF1	MluCI	T:10,17,25,69<br>A:10,17,44,25,25	Intron, A/T	ECA28	Rs396436853/<br>26198116	Wypchło et al. 2018
TGFβ	HinfI	T:203<br>G:93,110	3'UTR, G/A	ECA30	Rs782881835/<br>13922460	Wypchło et al. 2018

1. Dierks C. 2006- Molecular genetic analysis of quantitative trait loci (QTL) for osteochondrosis in hanoverian warmlood Horses. Dissertation. Hannover.
2. Komm K., 2010 – Fine mapping of quantitative trait loci (QTL) for osteochondrosisin Hanoverian warmblood horse. Dissertation. Hannover.
3. C.Wittwer, H.Hamann, O.Distl 2009 The Candidate Gene XIRP2 at a Quantitative Gene Locus on Equine Chromosome 18 Associated with Osteochondrosis in Fetlock and Hock Joints of South German Coldblood Horses) *J Hered* (2009) 100 (4): 481-486.
4. Wittwer C.E., 2006 – Mapping quantitative trait loci (QTL) and comparative analysis of positional candidate genes for osteochondrosis in South German Coldblood horses. Dissertation, Hannover
5. C.Wittwer, C. Dierks , H.Hamann, O.Distl 2008 Association between candidate gene Markers at a Quantitative Trait Locus on Equine Choromosome 4 Responsible for Osteochondrosis Dissecants in Fetlock Joints of South German Coldblood Horses. *J Hered* (2008) 99 (2): 125-129.
6. Wypchło, M., Korwin-Kossakowska, A., Bereznowski, A., Hecold, M., & Lewczuk, D. (2017). Polymorphisms of the COL9A2, AOAH and FRZB genes in the horse genome and their association with the occurrence of osteochondrosis.
7. Wypchło, M., Korwin-Kossakowska, A., Bereznowski, A., Hecold, M., & Lewczuk, D. (2018). Polymorphisms in selected genes and analysis of their relationship with osteochondrosis in Polish sport horse breeds. *Animal Genetics*, *49*(6), 623-627.
8. Lampe, V. (2009). Fine mapping of quantitative trait loci (QTL) for osteochondrosis in Hanoverian warmblood horses. *Hannover, Tierärztl. Hochsch., Diss*.
