# Supplementary material for: Connections Between Gene Polymorphism and Fetlock and Hock Measurements in Polish Sport Horses
Source: Int J Mol Sci. 2025 Oct 2;26(19):9645. doi: 10.3390/ijms26199645 (PMC12525504; doi:10.3390/ijms26199645)
Supplement: Supplementary file 1 [file ijms-26-09645-s001.zip › ijms-386161-Figures S1-S15.pdf]

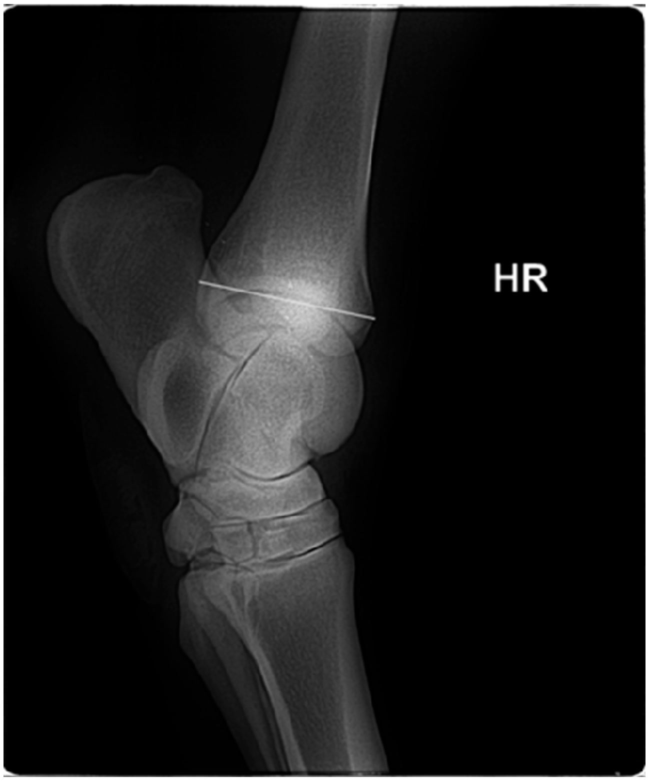

Figure S1. Measurement A - passes through the widest part of the distal epiphyses of the tibia and it is perpendicular to the long axis of this bone.

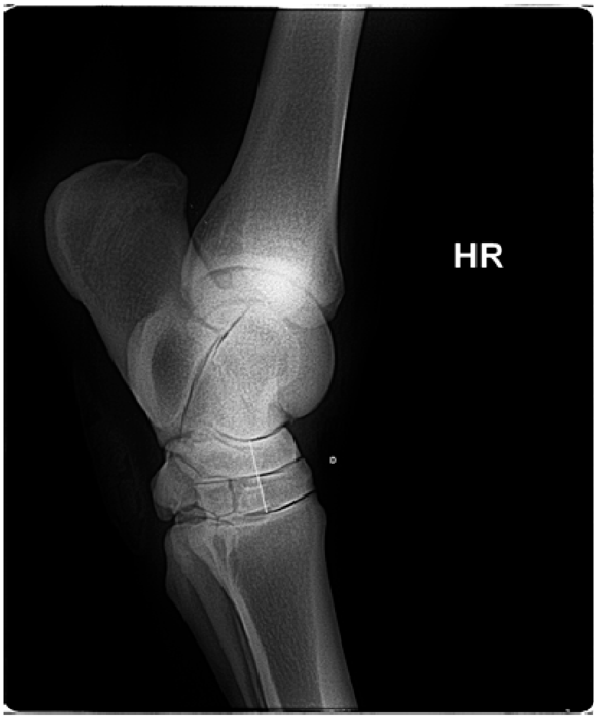

Figure S2. Measurement D - runs as an extension of the long axis of the 3rd metatarsal bone from the point on the central tarsal bone to the point on the 3rd tarsal bone.

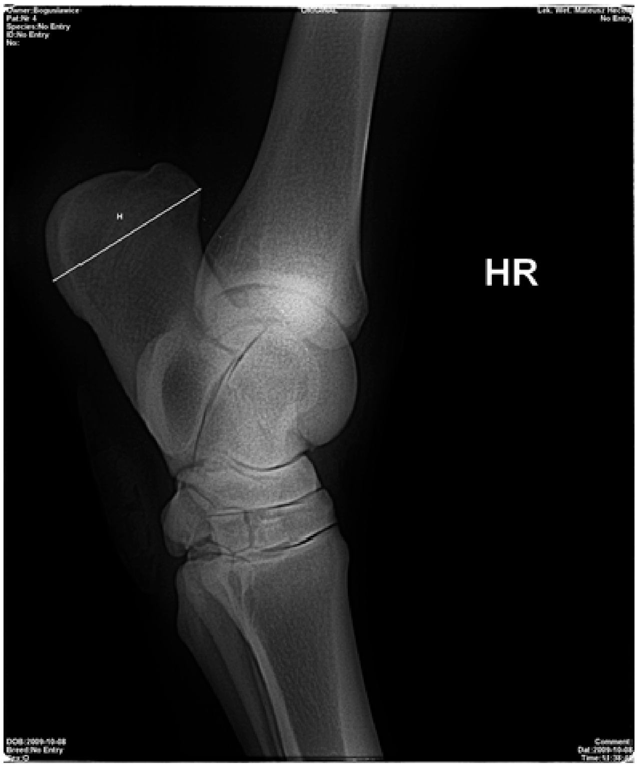

Figure S3. Measurement H - occurs at the widest part of the calcaneal tuberosity of the calcaneus from the outermost point of the dorsal side of this tuberosity to the outermost point of the plantar side of this tuberosity.

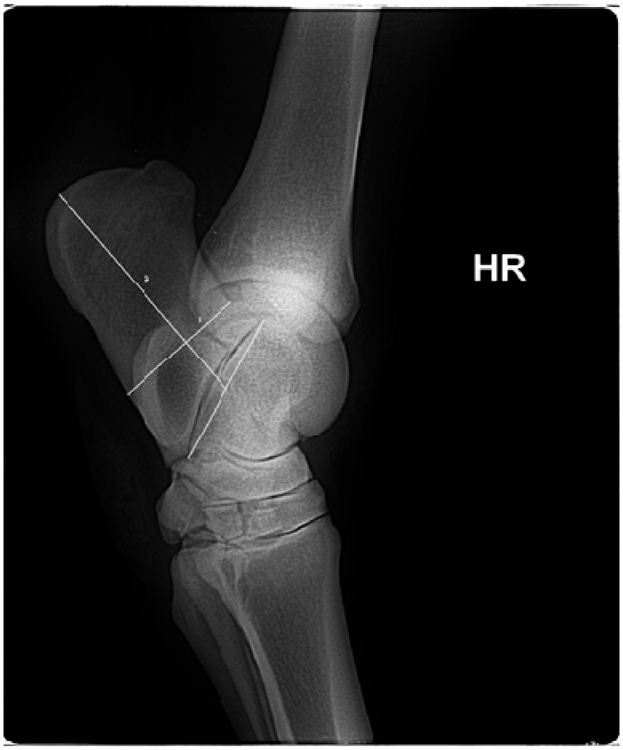

Figure S4. Measurement I - occurs at the narrowest part of the calcaneus from the outermost point of the dorsal side to the outermost point of the plantar side.

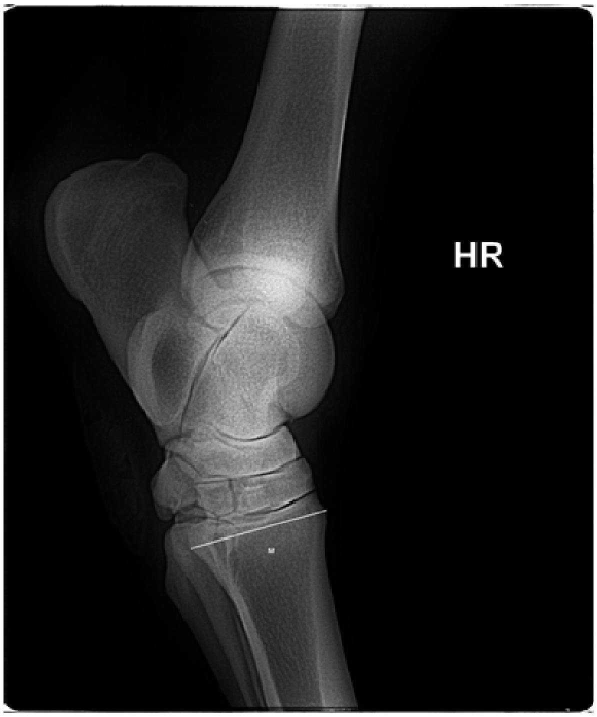

Figure S5. Measurement M - runs at the widest part of the proximal epiphysis of the 3rd metatarsal bone and it is perpendicular to the long axis of this bone.

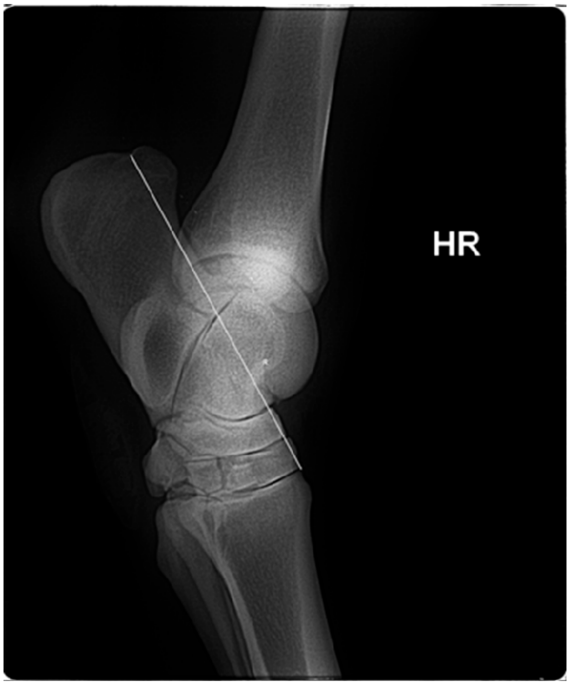

Figure S6. Measurement N - runs from the lowest point of the sagittal sulcus within the calcaneal tuberosity of the calcaneus to the starting point of the tarsometatarsal joint on the 3rd metatarsal bone.

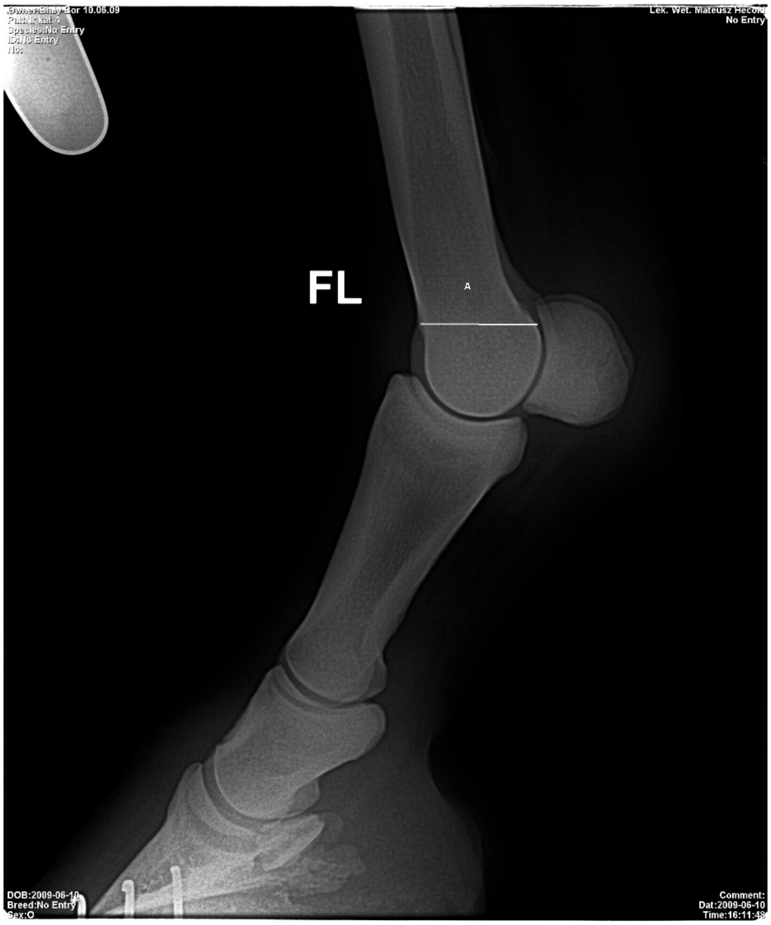

Figure S7. The line A from the beginning of the median crest of the distal metacarpal/metatarsal bone III to the beginning of the epicondyle.

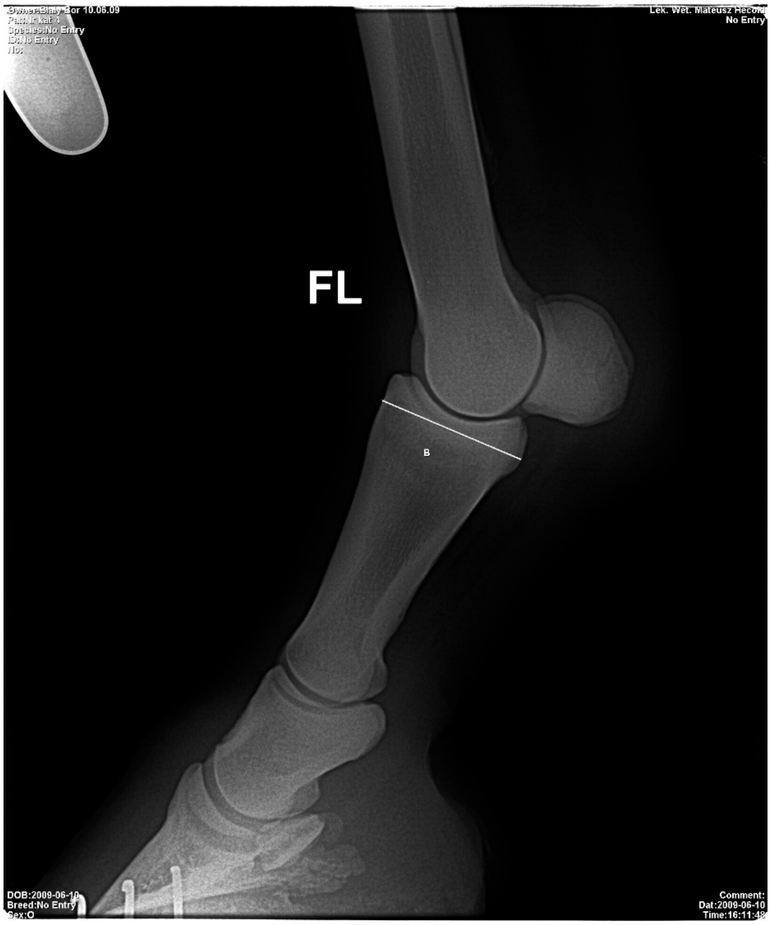

Figure S8. Dimension B includes the width of the proximal fetlock bone and runs parallel to the line connecting the two highest visible points of the joint surface of the fetlock joint, at its widest point.

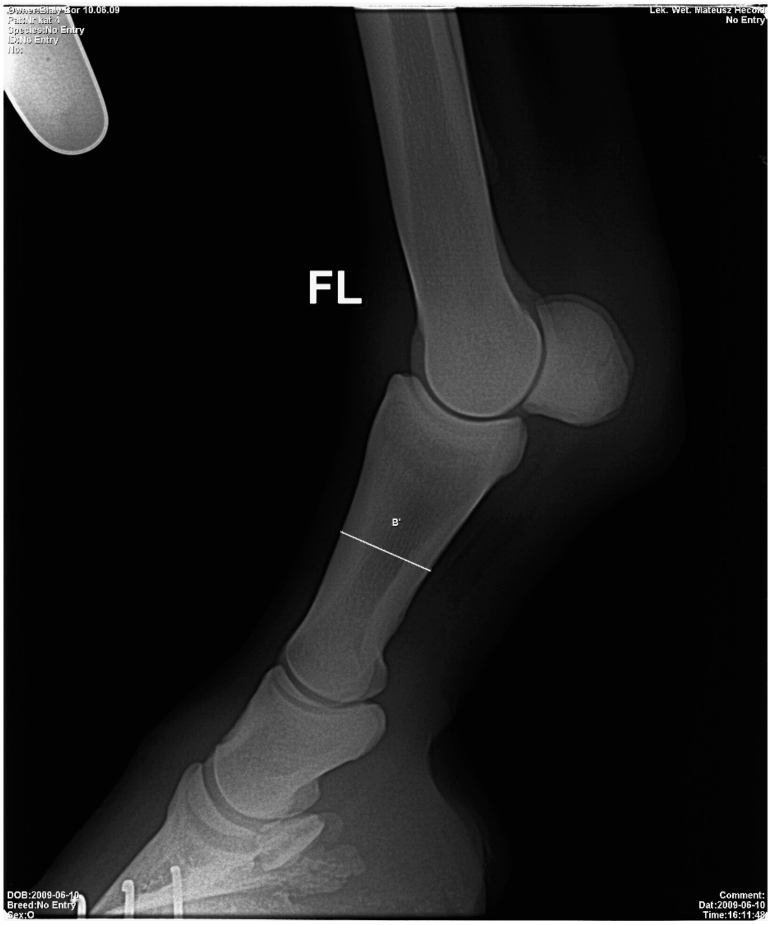

Figure S9. The dimension marked with the letter B1 runs halfway along the fetlock bone, perpendicular to the long axis of this bone, from the upper border of the dorsal cortex to the lower border of the ventral cortex.

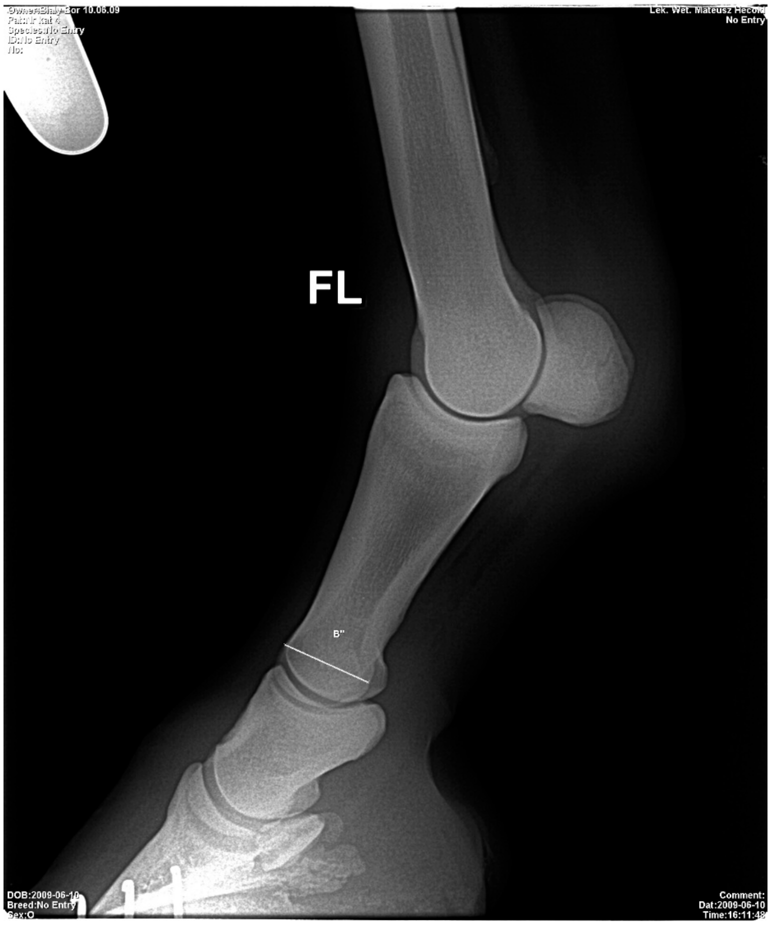

Figure S10. The dimension marked with the letter B2 is drawn parallel to the line connecting the two highest visible points of the proximal fetlock bone at the widest point of the distal fetlock bone.

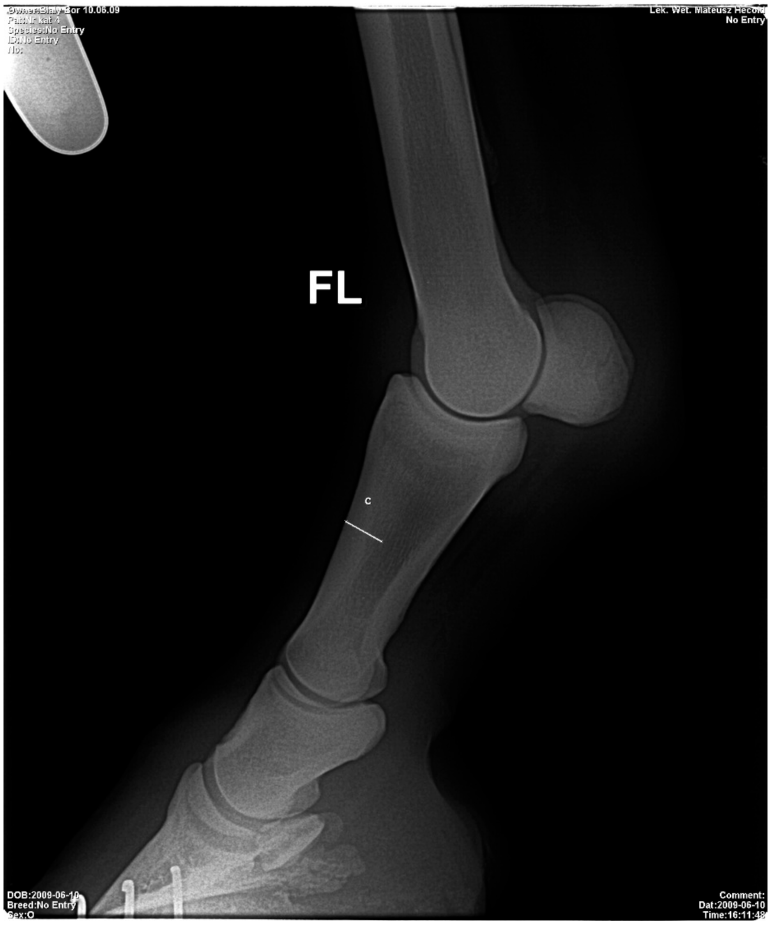

Figure S11. The dimension marked with the letter C determines the thickness of the dorsal cortex of the fetlock bone at the mid-length of the bone.

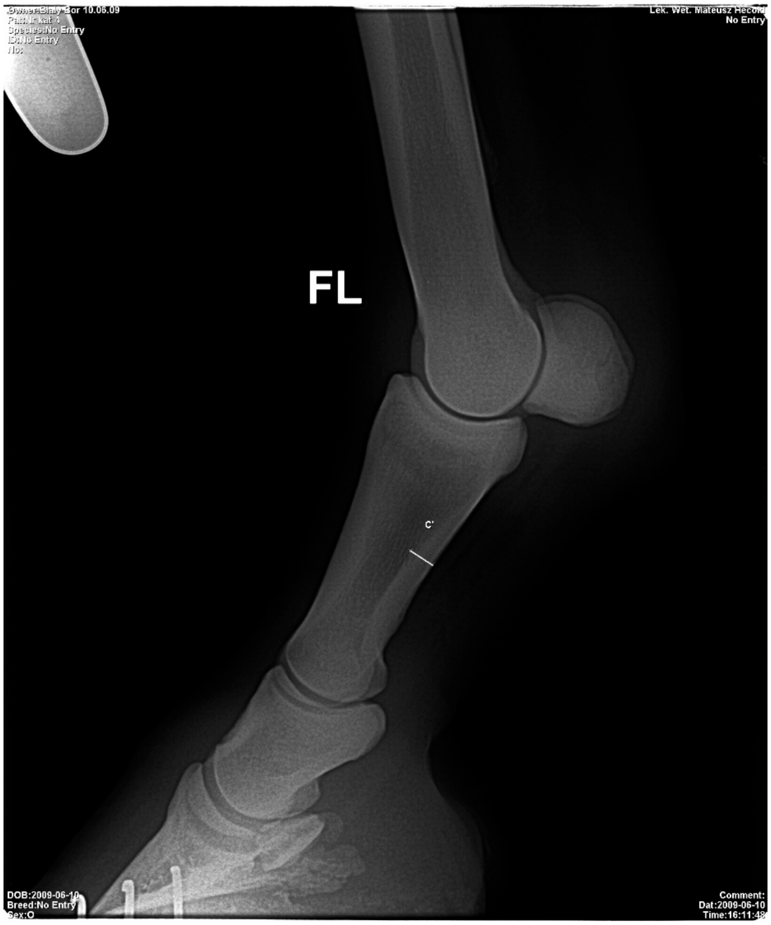

Figure S12. The dimension marked with the letter C1 determines the thickness of the cortical substance of the ventral surface of the fetlock bone at the mid-length of the bone.

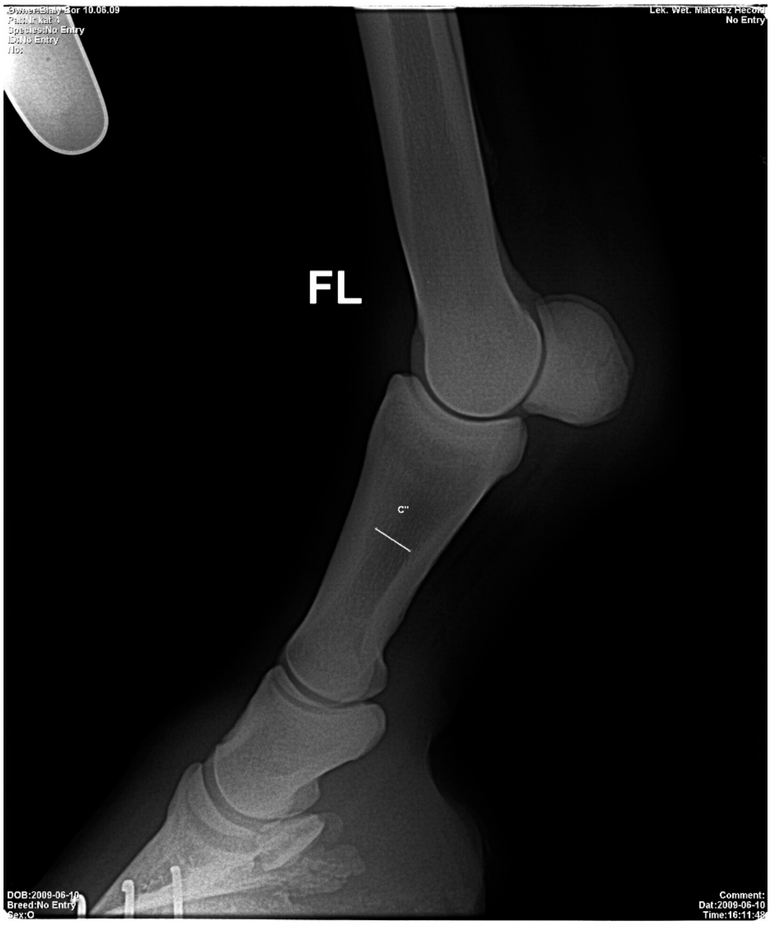

Figure S13. The dimension marked with the letter C2 denotes the distance between the inner borders of the dorsal and ventral cortex at the midpoint of the fetlock bone.

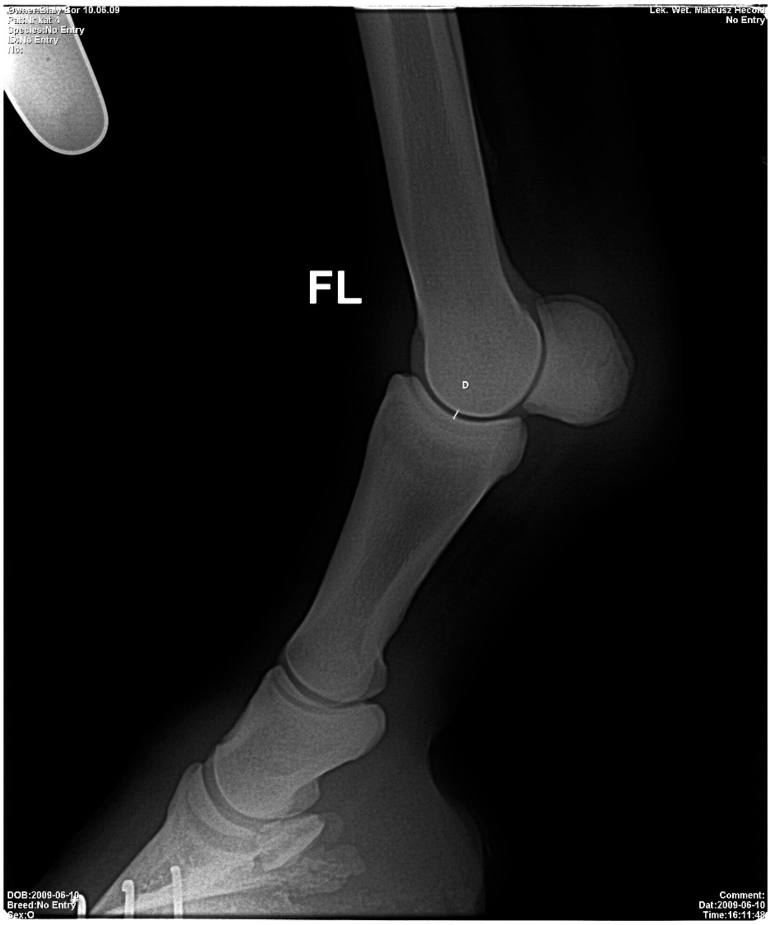

Figure S14. The dimension marked with the letter D indicates the width of the fetlock joint chink at its lowest point, measured from the surface of the distal metacarpal/metatarsal III bone to the surface of the proximal epiphysis of the fetlock bone. This section is aligned with the long axis of the fetlock bone

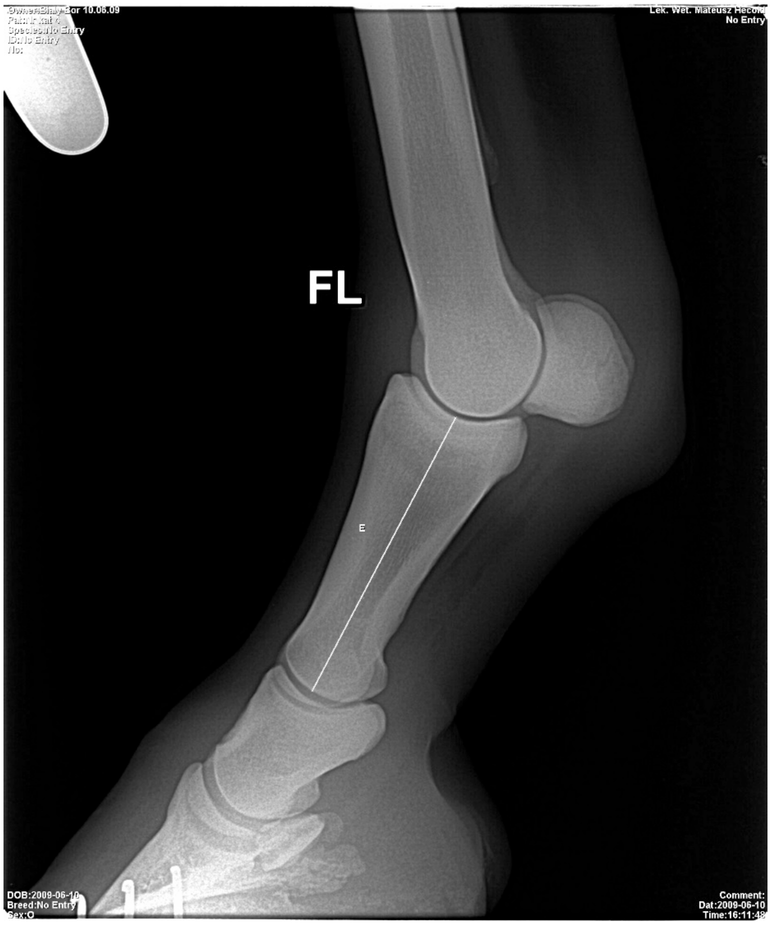

Figure S15. The dimension marked with the letter E indicates the length of the fetlock bone from the lowest point of the proximal epiphysis in the fetlock joint to the lowest point of the distal epiphysis of the fetlock bone in the coronal joint.
